# Set and Setting in the Santo Daime

**DOI:** 10.3389/fphar.2021.651037

**Published:** 2021-05-04

**Authors:** Ido Hartogsohn

**Affiliations:** The Graduate Program in Science, Technology and Society, The Interdisciplinary Studies Unit, Bar Ilan University, Ramat Gan, Israel

**Keywords:** psychedelics, hallucinogens, ayahuasca, set and setting, ritual, spirituality, santo daime

## Abstract

Set and setting is a fundamental concept in the field of psychedelic drug research, which points to the profound dependence of psychedelic effects on psychological, social, and cultural factors. Santo Daime is a Brazilian religion that makes elaborate use of ordering principles, techniques, and symbology to shape and direct the effects of the hallucinogenic brew ayahuasca. This paper systematically explores the tapestry of, inter alia, symbolic, social, cultural, psychological, aesthetic and musical elements that participate in the shaping of the psychedelic experience in the context of the Santo Daime ayahuasca religion. The methodically rich under the hood description of the mechanics of entheogenic initiation within the Santo Daime tradition provides a template for future studies of the role of context in psychedelic experimentation.

## Introduction

Recent years have seen a surge of interest in the therapeutic, spiritual, and creative uses of psychedelics. Commonly referred to as a “psychedelic renaissance,” current research into psychedelics has been primarily focused on the clinical potential of psychedelics. However, it is also characterized by the resurgence of a host of other uses and applications of these agents, including their use within spiritual, creative and recreational contexts (Langlitz, 2012; Brown, 2013; Pollan, 2018).

A recurring phenomenon in this new wave of research into psychedelics is a growing interest in the complex web of contextual, extra-pharmacological factors determining response to psychedelics. Commonly referred to as set and setting (Zinberg, 1984; Hartogsohn, 2017), the crucial dependency of psychedelic effects on this intricate web of relations has been a cornerstone of sociological, and anthropological research on psychedelics (See for instance Becker, 1967; Dobkin de Rios, 1984). It has also served as a theoretical and practical foundation for clinical analyses of these agents (Johnson et al., 2008; Carhart-Harris et al., 2018). Viewed as a whole, this diverse body of literature consistently and compellingly argues that the highly diverse effects of psychedelics are crucially determined by contextual factors.

The general principles of set and setting were first described in the early 1960s (Leary et al., 1963, Leary et al., 1964). Recent years have seen novel attempts to more closely examine and appraise the mechanisms and elements involved in shaping experiences with psychedelics (See for instance Studerus et al., 2012; Haijen et al., 2018; Kaelen et al., 2018). Dupuis describes the role of interpretative apparatuses in framing and shaping participant experiences at the Takiwasi ayahuasca center in Peru, terming this phenomenon “the socialization of hallucination” (Dupuis, 2020).

Despite its impressive growth, the literature on set and setting has so far lacked a comprehensive, rich and systematic account of the sonorously orchestrated extra-pharmacological elements that lend flavor and meaning to actual experiences with psychedelics.

This paper aims to provide such an account. It seeks to portray the intricate tapestry of, inter alia, symbolic, social, psychological, aesthetic and musical elements that participate in the shaping of entheogenic experiences in the context of the Santo Daime ayahuasca religion (henceforth referred to as SD). It explores the surprisingly intricate ways in which features of ritual environment conspire to elicit what are regularly described as profoundly religious, transformative, and cathartic experiences. Additionally, it discusses the types of tensions and challenges that this form of ritual practice tends to produce, and how these are negotiated by SD members.

One reason to focus the attention on the case of SD is that it presents the observer with a considerably formalized type of set and setting that is repeatedly enacted and performed on a mass scale and in diverse settings.^1^ Another important advantage of focusing on SD is that the practices of SD stand at the center of a substantial body of scholarly research, and that its social, historical and cultural contexts have been amply described. The SD set and setting thus provides a highly structured, carefully studied, example through which to examine the principles of set and setting. At the same time, as will be shown in the later parts of this paper, the set and setting of the SD it is also sufficiently dynamic and diverse to provide fruitful, thought-provoking exceptions and counterpoints that add depth and nuance to this examination.

This paper, then, provides a description of the set and setting conditions that shape experiences in SD rituals. It surveys historical, social and cultural contexts that have contributed to the emergence of these conditions and demonstrates how these complex contexts participate in creating a certain mode or style of entheogenic experience–the SD experience. Finally, complicating and adding nuance to this idea, it shows how the SD set and setting transforms across times and places, and how its typical dynamics give rise to several characteristic complications and challenges that SD practitioners are called upon to negotiate and resolve.

### The Concept of Set and Setting

The use of contextual cues to shape experiences with psychoactives has been prevalent in diverse cultures from times immemorial. Shamans commonly employ a variety of contextual methods to shape the experiences of their clients including ritual songs, smoke, whistles and sacred objects (Dobkin de Rios, 1984; Beyer, 2010). Sporadically discussed by anthropologists, drug enthusiasts and the occasional maverick psychiatrist (Mooney, 1896; Wallace, 1959; See for instance Moreau, 1973; Baudelaire, 1998), the role of contextual factors has generally been absent from psychiatric discourse of drug effects until the arrival of hallucinogenic research in the 1950s, which led to the first investigations in this direction. Mid-twentieth century writing on the role of extra-pharmacological factors noted the role of subject personality (Lasagna, 1963) of the clinician (Feldman, 1963), of physical and social setting (Dimascio and Klerman, 1960), as well as the expectations and intention of the individual, or the time of day, among other things (Eisner and Cohen, 1958; Leary et al., 1964).^2^ The term *set and setting* came into use in the early 1960s as a catch-all phrase for the psychological and environmental factors shaping drug effects. The term has been commonly used since (Hartogsohn, 2017).

The resurgence of psychedelic research over the past 20 years has been characterized by renewed interest in set and setting and attempts to gauge the relative significance of factors shaping psychedelic drug effects, under the assumption that the success of psychedelic practice is crucially dependent on the wise application of appropriate contextual cues (Johnson et al., 2008; Studerus et al., 2012; Carhart-Harris et al., 2018; Haijen et al., 2018; Kaelen et al., 2018).

Importantly, previous writing on Brazilian ayahuasca religions such as A Barquinha, União de Vegetal and SD put an emphasis on the fundamental role of ritual environment (Araújo, 2002; Cemin, 2004; Couto, 2004; Melo, 2016). The structure and form of SD rituals, have been detailed by authors including Cemin, Couto and Groisman (Groisman, 1999; Cemin, 2004; Couto, 2004). Most notably, the writing of Edward MacRae examined the ritual structure of SD through the prisms of controlled use and harm reduction (MacRae, 2008, 2009). Nevertheless, past accounts left much to be desired in describing the immediate and profound implications such ritualistic aspects have over member experiences, nor did they provide a systematic review of the varied aspects of set and setting, and the intricate ways in which these interact to produce a powerful pharmacologically and culturally induced response.

This paper seeks to fill this gap by providing a uniquely detailed, broad, and systematic analysis of the elements involved in the SD set and setting. It points to heretofore unacknowledged elements of SD set and setting, their complex interrelationships and implications. By presenting a thorough, methodic and rich under the hood description of the mechanics of entheogenic initiation within the SD tradition, this paper provides a template and a model for future studies that may seek to thoroughly and systematically examine other contextual environments and the characteristic ways in which they shape hallucinogenic response. It thus contributes to current attempts to gain a more integrative, cohesive and penetrating understanding of the crucial role of set and setting in shaping experiences with psychedelics.

### Brief Introduction to Santo Daime Religion and Its Set and Setting

SD religion is a Brazilian ayahuasca religion, which emerged in the Amazonian state of Acre in the 1930s. Founded by Raimnudo Irineu Serra (deferentially referred to by daimistas as Master Irineu), a black rubber tapper who emigrated to the Amazon region from the Brazilian state of Maranhão, SD developed throughout the mid-twentieth century, until 1971 and the death of its founder (Moreira and MacRae, 2011). Following this event, the church split into several distinct lines. One of these lines, led by Sebastião Mota de Melo (deferentially referred to as Padrinho [godfather] Sebastião), a disciple of Master Irineu, went on to establish itself across Brazilian urban centers, and eventually became internationalized with centers in Europe, Asia, Australia, North and South America.^3^ In order to delimit the scope of this study, this paper concerns itself exclusively with Sebastião’s line which achieved national and global prominence since the breakup of the original church in the 1970s. Formally known as ICEFLU (*Igreja do Culto Eclectico da Fluente Luz Universal*), this line will here forth be identified simply as Santo Daime (or SD).

At this point, I’d like to briefly introduce several key terms crucial to any description of SD religion. At the center of SD religion stands the ritual consumption of the ayahuasca beverage (referred to as *daime*) in a carefully organized context, centered around the joint musical performance of ritual hymns (*hinos*), within a ritual space referred to as the *salon* (*salão*). SD rituals are collective endeavors performed by congregations (churches), and referred to as works (*trabalhos*), a designation which signifies the arduous, physically and psychologically demanding nature of SD rituals. During such works, members believe to be receiving visions and guidance from the *astral*–a higher order spiritual realm, which becomes accessible under the force of daime. SD works take place under the more general symbolic and religious framework referred to as “the doctrine” (*doctrina*), a ‘multi-vocal’ term (Groisman and Sell, 1995, 250), which is invoked repeatedly despite never being properly defined, but generally points at the fundamental spiritual and metaphysical principles underlying and validating SD practice.

My analysis of the set and setting of SD will be focused on standard SD ceremonies,^4^ and based on the key factors from the set and setting model I described elsewhere, with adaptations (Hartogsohn, 2015).

I begin by exploring the aspect of set, looking first at the role of preparation, expectation, and intention. I then move on to describe the setting of SD works, focusing first on the physical environment and the diverse ways in which the SD setting engages sense organs such as sight, sound, smell, proprioceptive and kinetic senses. My examination continues with the social aspects of the SD ceremonies by considering the many ways in which the communal aspects of SD religion shape the experiences of daimistas. Expanding on the classic model of set and setting, this analysis employs two additional dimensions of contextual elements–the skillset which ritual participants acquire and utilize during SD works, and the dimension of post-session integration.^5^

## Set: Preparation, Expectation, and Intention

### The Divine Origin of Ritual Set and Setting

A fundamental and distinguished feature of the SD tradition is that it regards the very structure of ritual–the set and the setting–as a sacrosanct product of divine revelation.

The divine status of the SD set and setting is traceable to the foundational story of Master Irineu’s encounter with the Queen of the Forest, whom Irineu immediately recognized as concurrently representing the Christian virgin of Conception (Moreira and MacRae, 2011, 87–102). According to Moreira and MacRae, “among the lessons conveyed to the young Irineu by this feminine instructor at the beginning of his initiation was the renaming of the terms used by the Indian and Mestizo vegetalists to identify the drink, the plants and the effects that they produce” (Moreira and MacRae, 2011, 100). The recalibration of language, and therefore symbolical framework, or set, was thus part of Irineu’s original revelation. Orgad writes that Irineu’s revelation could be regarded as a “penetration of the Gods–in this case the Virgin Mary–into the world in a way that reinstituted the ‘sacred doctrines.’ This institution has brought with itself a new communitarian structure, a new religious calendar and a new ritual” (Orgad, 2012, 96). Irineu’s revelation can thus be recognized as a seminal moment in which the foundational elements of the SD set and setting are received from divine origin.

Over the next decades, the ritualistic repertoire of SD evolved and developed under the direction of Master Irineu, achieving sacrosanct status through the established position of Irineu as a prophet and reincarnation of Jesus Christ within daimista faith. This period saw the emergence of the concentration ritual, the basic ritual of the SD doctrine, and the evolution of other types of works such as healing works, mass works, and hymnary works.^6^ Remarkably, the alterations and adaptations in ritual styles became increasingly energetic and dynamic during the last decade of Irineu’s life (Moreira and MacRae, 2011, 305–334).

Importantly, the development of novel ritual structures, does not stop with Irineu’s death. Following his passage, the question of proper set and the setting became a central point of contention in the ongoing strife between Irineu’s original center in Alto Santo, and the ICEFLU line which soon became disseminated in many other parts of Brazil.^7^ Under Padrinho Sebastião’s direction, SD repertoire continued evolving. Sebastião recreated the format for concentration and healing works and added new types of works such as White Table (*mesa branca*) works. These innovations were complemented by the incorporation of spiritist and Umbanda elements into daimista ritual and the integration of the ritual use of cannabis (renamed Santa Maria) into the doctrine (MacRae, 2008; Dawson, 2013). Crucially, these developments represented a significant break from the practices of the Alto Santo group, which strove to retain the ritual structure left by Mestre Irineu, and viewed it as finalized and non-alterable (Orgad, 2012).

Thus, while one of the hymns by Padrinho Alfredo, son of Padrinho Sebastião and current leader of ICEFLU (who himself introduced a new type of ritual–the St. Michael work) states “really following the doctrine/not changing even an tilde,”^8^ any examination of the history of SD and its ritual reveals a multi-layered, palimpsestic quality. Such a development harks back to indigenous and local forms of ritual, adapted by Master Irineu, reaching a third stage of development at the hands of Padrinho Sebastião and his followers, while a fourth stage of adaptations is currently taking place owing to the spread of SD into urban centers in Brazil and abroad, as observed by Dawson (Dawson, 2013).

Despite the dynamic and diverse nature of SD ritual, a fundamental communality of principles, regulations and conventions remains at its core.

It is therefore interesting to mention one notable attempt for the standardization and codification of SD ritual, which occurred at the hands of Padrinho Alex Polari, a writer and ex-guerilla fighter who spent years imprisoned by Brazilian military dictatorship, and later became a SD community leader (Alverga, 1999).

In 1997 CEFLURIS (former name of ICEFLU) published an official document written by Polari with the title *Norms of Rituals* (*Normas de Rituais*) sanctioned by leading SD figures with the intent of creating an established guide for the conduct of SD rituals (CEFLURIS, 1997).

*Norms of Rituals* is by no means meticulously followed by SD churches, and it is certainly not read by all or even most SD church members, yet it presents an interesting example of an attempt to codify the set and setting of SD rituals. It can be consulted in cases of doubt and serve as a useful guidepost.

In practice, few churches follow all the instructions contained in *Norms of Rituals*. Importantly, however, *Norms of Ritual* leaves space for such deviations. It begins with the caveat that even the sacred principles of ritual cannot be allowed to fossilize and become an obstacle. Rather, it argues, ritual should be adapted to changing circumstances and conditions. *Norms of Ritual* should therefore be viewed as an attempt to lay out general principles of SD ritual rather than set them in stone.

Despite this commitment to adaptability and flexibility, SD ritual prescriptions remain a central part of the doctrine. What does it mean when the set and setting for hallucinogenic experiences are consecrated and meticulously defined? To begin with, it means that in comparison with most other types of set and setting, SD members are driven by a defined sense of purpose, that of serving the holy ordinances of ritual. Their actions in ritual space thus (ideally) transcend their individuality, allowing them to become servants of a higher order of meaning manifesting in the minutest details of ritual observance (as well as in the fluidity and flexibility necessary for the observance of the ritual’s spirit).

### The Sanctity of the Santo Daime Beverage

Another framing factor that shapes SD experiences is the sacred status accorded to the daime beverage itself (Couto, 2004). The name *Santo Daime* (translatable to ‘sacred give-me’) was received by Irineu in his initial mystical encounter with the Queen of the Forest. Thus, the name’s origin story and literal meaning both point to its sanctified status within the SD. Daime is to be used exclusively in a manner conforming with the prescriptions of the doctrine (e.g. with preparatory sexual and alcoholic abstinence), and the meticulously prescribed and ritualized manner in which the daime beverage is prepared during SD *feitio* (beverage preparation) rituals serves to distinguish it symbolically from otherwise chemically similar preparations of ayahuasca. Daimistas make a point of using only daime (as opposed to non-SD originating ayahuasca) in their rituals. Daime is not ayahuasca, ayahuasca is not daime.

The daime beverage is accorded a supreme spiritual status. By drinking daime one is said to drink the blood of Christ, making it at least symbolically similar to the Catholic sacrament (Cemin, 2004). In serving hymns (*hinos do despacho*), a selection of SD hymns extolling the sacred beverage, daime is described as a ‘divine being transformed into a liquid,’ (Silva J, 45), as a “teacher of all teachers” (Melo Alfredo, 84), and even as “the divine eternal father and his son the savior” (Melo Alfredo, 84).

The effects of daime are likewise eulogized as intrinsically favorable. Daime is reputed to heal, instruct, and purify the individual and the community. It is drunk, with good faith, under almost all circumstances: in pregnancy, in birth and in baptism, in health and in sickness. Its effects are regarded as universally benign.

This belief in daime as a superior, benign being is crucial to the shaping of the SD experience. One might tremble before drinking daime, yet the devout daimista rests assured in the knowledge that they are in the good hands of the “divine father and his son the savior”, of “the teacher of teachers,” who wishes them well and has infinite intelligence and power to heal and instruct. This added layer of trust in the beverage fortifies the daimistas in their struggles, cushioning their sorrows in the reassurance of divine protection.

### The Symbolical World of Santo Daime

Experiences with psychedelics are deeply shaped by the surrounding cultural environment (Wallace, 1959; Hartogsohn, 2020). A central part of this cultural web is the symbolic network of figures, motifs, and patterns that are emblematic of cultures. In SD this network is crucially mediated by the textual contents of SD hymns which unfold throughout the works on paricipants’ mouths.

SD’s symbolical universe is notably diverse, as befits the remarkably eclectic character of the SD doctrine, acknowledged in the in the very acronym of SD head organization ICEFLU–Church of the Eclectic Center of the Flowing Universal Light.

The list of cultural sources from which SD ritual draws is extensive. One such source is the indigenous, vegetalista traditions, which were familiar to Master Irineu and inspired diverse ritual elements including the use of dietary restrictions, the references to forest plants and animals (particularly the hummingbird), and the use of spirit calls (*chamados*) (Macrae, 1992; Dawson, 2013). The São Paulo based Esoteric Circle, with which Master Irineu was associated in the early days of the doctrine also exerts notable influence through the integration of “alternative ‘philosophies’ and non-mainstream ‘scientific’ world views” (Dawson, 2013, 16), and through the ritual inclusion of texts originating from the group (e.g. ‘consecration of space,’ and ‘key of harmony’) (Dawson, 2013). A spiritist influence, introduced through Padrinho Sebastião’s involvement with the Allan Kardec's tradition of spiritism is evidenced in the central role of mediumship work within the ICEFLU line, and the integration of the entities and spirits of Brazilian spiritism, such as Doctor Jose Bezerra de Menezes and Professor Antônio Jorge (Macrae, 1992, 3; Dawson, 2013, 23). Similarly, the influence of the Afro-Brazilian religion Umbanda, integrated into the doctrine in the 1980s, can be recognized in the inclusion of Umbanda entities such as Ogum, Oxum, Oxalá, Yemanja, Xangô and Exu (Dawson, 2013, Ch. 1). Additional sources of influence can surely be added to this list. However, the most prominent is surely the influence of Christianity, specifically folk Catholicism (Dawson, 2013, Ch. 1). The protagonists of Christian religion figure prominently in SD cosmology and hymns, including the Archangels St. Michael and St. Raphael, St. John, St. Joseph, the Virgin Mary, the holy spirit, the eternal father, and of course Jesus Christ himself. Complementing this Christian assembly of deities and saints are frequent references to the natural world: the sun, the moon, the stars, the sea, the forest, the sky, and ‘nature.’ The accompanying semantic and symbolic significations of these remarkably diverse entities and beings all add distinct flavor to SD works and experiences.

Additionally, one needs also pay attention to the conceptual language used by daimistas to describe their rituals. Dawson describes the designation of rituals as *works* as a form of “symbolic regulation through which the ‘obligations’ implicated in daimista ritual space are driven home” (Dawson, 2013, 65). Similarly, when daimistas speak of an energetic ‘current’ (*corrente*) that imbues the work with ‘force’ (*forca*)*,* this provides a certain framework to ritual. When they sing of being “inside the force” (Serra, 89), or “inside the battle” (Germano, 29), or when they profess their faith that “the force of this world/it exists certainly” (Maria, 33), and that this force is the sole director of reality, this has profound influence on daimistas mindset and their conceptualization of ritual events, shaping their response and attitude. Moreover, frequent reference to lofty values and ideals such as love, faith, truth, harmony, justice, and forgiveness (alongside references to misery and the illusory nature of this world) also inescapably shapes the types of visions and inner processes daimistas experience under the influence of daime. Finally, SD hymns make recurring reference to a celestial mother and father. Members repeatedly sing of seeking their father’s love and their mother’s comfort, and of trying to be good brothers and sisters. Such recurring familial references have profound impact and facilitate intensive engagement with one’s own familial relationships, which daimistas often report.

SD symbology is rich and an extensive review of the symbolical web of references in SD is well beyond the scope of this paper.^9^ This short exposition should thus be seen as a preliminary introduction to a subject that merits a separate, more thorough, examination.

### The Holy Calendar

Daime works are held at dates specified by the SD calendar. Alongside the habitual concentration work, which take place each 15^th^ and 30^th^ of the month, the most crucial of these dates are dancing works (*bailados*) which are held in specified dates of religious significance derived from the Christian calendar (e.g. day of St. John, the day of St. Peter, Christmas, the Three Kings Day), or from the history of SD’s lineage (e.g. the birth and death dates of Master Irineu and Padrinho Sebastião).

Holding the works at specific dates charged with religious significance shapes mental set. The fact that works are held annually, in specific dates commemorating significant religious events, and that the works are held simultaneously at numerous locations worldwide, lends them bigger-than-life global and cosmic proportions. One could think of these events as representing the date of a holy battle which is reenacted by the participants at the divine mandate of the doctrine. This fits well with Mircea Eliade’s concept of sacred mythical time, as a moment, outside history, in which the sacred is allowed to penetrate into profane reality. In *Myths, Dreams and Mysteries* Eliade writes: “In imitating the exemplary acts of a god or of a mythic hero, or simply by recounting their adventures, the man of an archaic society detaches himself from profane time and magically re-enters the Great Time, the sacred time” (Eliade, 1986, 23). By participating in SD ritual held at significant dates, participants are transported outside everyday reality and into a mythical realm where their ritual actions are synchronized with events of mythic proportions. Ritual time thus “breaks the linearity of normal time and brings us back to a time of synchrony” (Couto, 2004, 400).

### The Role of Astral Militarism

“I entered into a battle/I saw my people weaken/We have triumph/With the power of God” (Serra, 115).

A prominent aspect of the SD doctrine that demands an explanation is a striking tone of what might be termed astral militarism (Groisman, 1999; Dawson, 2013). SD language is replete with military references to battles, phalanges, enemies, soldiers, arms, swords, shields and medals. Most notably, Master Irineu is regularly referred to as the General Juramidam, the different sections of daimistas dancing in the ritual space are called battalions, members wear uniform marked with the SD insignia (the six pointed star), and the leaders of SD church are referred to as commanders (Couto, 2004, 395). The emphasis on discipline, uniforms and organization in lines is also reminiscent of military form. Originally, such elements took on an even more central place in the ritual. SD uniform originally included military-like hierarchical ranks, and military salutes were exchanged between members whenever entering or exiting their ritual positions (Couto, 2004; Dawson, 2013).

Some researchers have historically attributed these motifs to Irineu’s experiences as a soldier in the Brazilian territorial guard (Mórtimer, 2019), while others have pointed to the ubiquity of combative language and metaphors in shamanism and the rituals of *pajelança* prevalent in Irineu’s ancestral land of Maranhão (Couto, 2004; Labate and Pacheco, 2004).

The concept of battle (*batalha*) is key in this regard. SD rituals are understood as spiritual battles taking place in the salon, and in the hearts and minds of participants. Participants are admonished to stop the phalanges of Lucifer from infiltrating the salon (Melo Valdete, 12), and urged to fight the battle of love (Melo Alfredo, 132).

Such militant language might startle some individuals with pacifist sensitivities. Nevertheless, SD members generally perceive this astral militarism as fundamentally benign and life-affirming, as showcased in concepts like “army of light” (Froés, 47), or “sword of forgiveness.” (Melo Alfredo, 25). Military language is used to unify, motivate and energize daimistas as they enter into long and arduous spiritual battles.

This often arduous nature of SD works can be recognized by their designation as trials (*provas*) (Dawson, 2013, 64–65). Dawson remarks that “the physical demands of often prolonged ritual participation predicated on sustained coordination with collective ritual dynamics makes for a doubly demanding experience” (Dawson, 2013, 65), and sums up: “Daimista ritual space is no place for the faint-hearted” (Dawson, 2013, 65).

Crucially, withstanding the daunting trials of long and formidably arduous SD works/battles is thought to assist in the development of broader abilities useful for confronting the challenges of everyday life. Ritual work is here understood as a sort of spiritual bootcamp that allows participants to cultivate useful cultic virtues such as strength, joy and firmness–the ability to withstand difficulties while staying centered and anchored in divine love (See for instance Melo Sebastião, 3). This spiritual ideal is capitulated in the lines “I am inside the battle/Suffering but happy” (Melo Rita, 25), sung at the end of SD works. The dramatic battleground of SD introduces great pains but also to immense joys, in accordance with the description of the psychedelic experience as a space of intensified experience (Hartogsohn, 2020). Here, in other words, is the crucible of one's spiritual battle.

The concept of battle is crucial to understanding daimista expectation and intention. Unlike many other sets and settings for psychedelic use, which recommend letting go and surrendering to the experience–daimistas commonly walk into the daime ritual galvanized and ready to partake in ‘spiritual warfare.’^10^

### Preparing for a Work

Works about set and setting often recommend dedicating time, attention and intention to prepare before a psychedelic experience (Leary et al., 1964; Godasi, 2019). Dedicating time and injecting ritualistic elements such as diets, sexual abstinence and prayer in the days leading to a ritual help orient a person towards a meaningful experience (Tramacchi, 2004). The key point is commitment, and as observed by MacRae, the specifics of shamanic diets are often of lesser importance than their ability to challenge established routine and assist in amplification of intention (Macrae, 1992, 6). SD ritual protocol, similarly to those of many shamanic traditions, prescribes a period of sexual abstinence before and after a work, and a period of abstinence from alcohol (Macrae, 1992). In some cases, members might take on specific forms of diets. Master Irineu famously undertook a manioc-only diet during his period of initiation (Moreira and MacRae, 2011, 1). Prayers are regularly uttered immediately prior to the beginning of a work. In some cases, prayers are performed in the days leading up to a work. Members might, for instance, perform a *Novena* (9 consecutive days of prayers) or *Quaresma* (40 consecutive days of prayers) in the days leading up to a St. Michael healing work, thereby priming themselves for a meaningful experience. The literature on classic (5-HT2A agonist) psychedelics (that include ayahuasca) emphasizes their ability to induce religious and mystical-type experiences (Pahnke and Richards, 1966; Griffiths et al., 2011). Such mysticomimetic potentialities of ayahuasca are thus catalyzed and augmented through above-mentioned contextual elements like holy calendar, sacrosanct status of daime, and ritual preparation.

### Identity

Issues of personality and identity have received some attention in research on set and setting. Early psychedelic research proposed that personality structures play a large role in determining therapeutic outcomes (Sandison, 1956; Eisner and Cohen, 1958), while more recent writing focused on identity (Saldanha, 2007; Cavnar, 2014; Devenot, 2016; Neitzke-Spruill, 2020; Williams et al., 2020).

Leaving aside the complex question of personality, issues of identity are clearly relevant to shaping the experiences of daimistas. SD space is strictly divided between the male and female, who are kept separate, confined to their respective zones.^11^ This separation is explained as a separation of the male and female ‘currents’ or ‘energies.’

Gender identities are also basically inflexible. Members wear distinctly gendered uniform (skirts and crowns for women, suits and ties for men) and the virginity and marital status of members is marked by specific items (palm and flower) adorning their attire. Furthermore, ritual functions follow traditional gender roles. Males are responsible for guarding the entrance to ritual space and making celebratory ‘Viva’ calls. Women, by contrast, are responsible for leading *terço* prayers, preparing the flower arrangements and filling the role of lead singer (*puxadora*). Finally, the *feitio*, the complex ritualistic process of daime preparation, is strongly governed by gendered roles. The collection and preparation of the DMT containing *chacruna* leaves (understood as representing the feminine side of daime) is performed by the women, while the gathering and preparation of the jagube vines (understood as representing the male side of the daime) are handled by the men (Macrae, 1992, Ch. 3).

This division of participants into two distinctly gendered sections has profound implications for participants. The women section and male section of a church are often quite different. In some churches, one is more dominant than the other. More generally the male and female battalions tend to have distinct characters, often reflective of their gender, so that men and women are embedded in different types of environments during the ritual.

The strongly delineated gender roles of SD also complicate the inclusion of non-cisgender non-binary individuals into SD rituals. The appropriate positioning of such transgender or non-binary individuals in ritual space is thus contestable (Hartman, 2019). And while some LGBTQ daimistas have claimed that SD rituals help them reaffirm their sexual identity (Cavnar, 2014), the sexually conservative character of SD might be considered non-inclusive or even repressive by members of the LGBTQ community (Dawson, 2013; Hartman, 2019).

## Setting–Physical, Social and Cultural

### Order in the House: The Arrangement of Space in Santo Daime Rituals

Couto defines SD as a ritual of order (*rito da ordem*)*,* highlighting the regulated, structured nature SD ritual space (Couto, 2004). This becomes easily recognizable in Dawson’s succinct account of SD space:

Daimista ritual space is most commonly organized hexagonically, with one half of the floor occupied by male participants and the other by female practitioners. … the male and female battalions are divided into three sections … these sections are oriented as spokes around a central hub occupied by a table; preferably, though not always, shaped as a six-pointed star (the esoteric ‘Star of Solomon’) … Most Santo Daime rituals are undertaken with participants facing inward towards the ‘star-table’. The star-table is commonly laid with ‘the cruzeiro’ (i.e. two-sparred cross) draped by a rosary, statuettes of Mary and Jesus and photographs of significant figures such as Master Irineu and Padrinho Sebastião. Representing the elemental forces of earth, water, wind and fire, the table is also laid with flowers, a jug of water, incense sticks and candles. (Dawson, 2013, 46–47).

**FIGURE 1:**
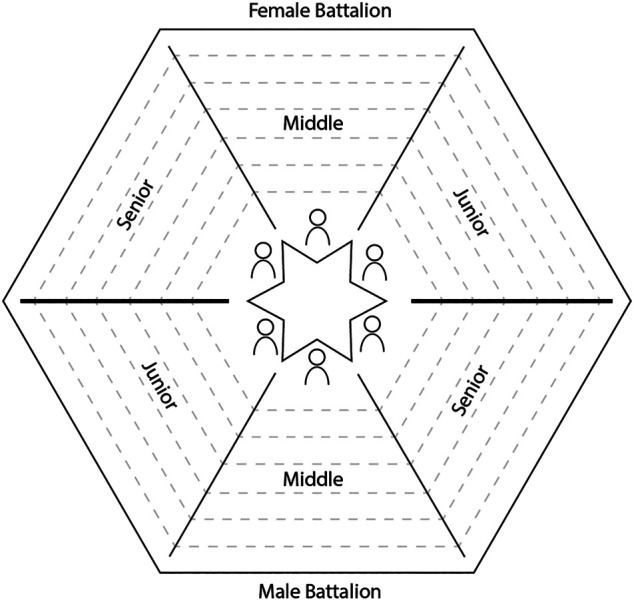
Spatial arrangement of SD ritual.

The noted hexagonal arrangement (see Figure 1) is significant on several levels. On the visual, aesthetic level, it represents a symmetrical, visually pleasing design (Hartogsohn, 2018b). On an auditory level, placing members facing each other in a closed form enhances acoustics. On a symbolical level, the mandalic organization of space is thought to reflect the divine order of ritual. On the level of proxemics (the study of space and its use) (Hall, 1990), the drawing of personal space and discouragement of physical contact is reaffirmed by the division of space between participants both in sitting works (where participants are assigned designated sitting spots) and in dancing works (where movement is delimited by marks on the floor). Socially, SD ritual space places the participants facing each other, adding not only to visibility and audibility, but also to emotional intensity and the sense of mutual involvement. Space is also determinant of social and ritual hierarchies. Dawson adopts a Foucauldian perspective and expounds three disciplinary methods that establish SD ritual as “a disciplinary regime oriented to the maintenance of physical and symbolical order” (Dawson, 2013, 45). Notably, Dawson describes the spatial ordering of ritual environment as “field of power” (Dawson, 2013). Participants are typically positioned in accordance with their status in the group.^12^ Those of higher standing are closer to the center than those of lower standing. This positioning is not only of symbolic importance. Rather, as Dawson points out, it also carries its spiritual benefits for those well positioned. Such benefits include healing, purification or self-understanding facilitated through a position close to the ritual table and the center of the current (Dawson, 2013, 69–70).

### Visual Components

The role of imagery in healing and placebo has been noted by numerous scholars See for instance; (Ulrich, 1984; Achterberg, 2002; Case et al., 2018). Owing to the intricate entanglements of mind and body, a pleasant, reassuring view easily translates into a meaning response mediating placebo effects and healing (Hartogsohn, 2016). Arrangement of space is also described as paramount in therapeutic and spiritual work with psychedelics. Within the amplified experience of psychedelics, any minute detail, beautiful or ugly, might induce strong subjective responses. Some authors criticized the dismal effects created by the bleak environments of early psychiatric investigations with psychedelics (Lee and Shlain, 1992), while others offer guidelines, and labor to develop carefully arranged environments that induce optimal therapeutic effects (Johnson et al., 2008; Fagerbrink et al., 2019; Godasi, 2019).

The impressive effects of classic psychedelics on visual perception and their ability to induce visions and enhance aesthetic appreciation have been long reported (Ditman et al., 1962; Leary, 2007). SD religion utilizes such enhanced visual impressibility to facilitate transcendental experiences of beauty and harmony. Returning to Dawson’s description of ritual space, one easily recognizes the symmetrical arrangement of space, conforming with evolutionary inherited standards of beauty which show strong preference to symmetric features (Rikowski and Grammer, 1999; Penton-Voak et al., 2001). This visually pleasing hexagonal structure is further embellished using colorful decorations, as per the principle of ritual adornment in entheogenic ceremony mentioned by Tramacchi (Tramacchi, 2004).

Next, one needs to pay attention to the visual and symbolic qualities of the star table–set with elements that are juxtaposed carefully and in an aesthetically pleasing way, in order to elicit feelings of awe and reverence. Table arrangement is performed by select, skillful individuals and involves not only the use of visually pleasing objects such as flowers, colorful icons, crystals, and glowing candles, but also by the calculated use of symbology–syncretically adjoining both Christian symbols such as the two-armed Caravaca Cross and non-Christian symbols of ecumenical appeal representing the four elements (candles for fire, crystals and stones representing the earth, incense symbolizing the air, and glasses filled with water). To these are added images of the masters of the doctrine, Master Irineu, Padrinho Sebastião, Madrinha Rita and other trusted figures.

It is hard to overstate the importance of this imposing presence at the heart of the SD ritual space. This centripetal hub of ritual activity serves as an altar towards which the daimista turns physically as well as spiritually. Carefully arranged as a symbolically laden border zone for liminal transactions between the human and the celestial, the star table receives the prayers of participants whilst radiating beauty enhanced by the effects of daime. Its visually appealing, symbolically laden landscape serves to plant and reaffirm faith and hope in the hearts of participants facing it throughout the ritual. For those seated at the table, its aesthetically pleasing and symbolically inspiring contents occupy the field of vision almost entirely, acting as a persistent reminder of the doctrine’s spiritual ideals, and mitigating internal tribulations through reaffirming visual cues.

Another important visual element is the daimista ritual uniform (*farda*). The use of ritual uniform is common to the three ayahuasca religions of Brazil, deriving from traditional Catholic festivals (Labate and Pacheco, 2004; Dawson, 2016, 67–68). SD members use two types of uniform in their ritual. The blue uniform (*farda azul*) is used for seated works including concentration and healing works, while the white uniform (*farda branca*) is reserved for celebratory occasions.

The blue uniform*,* introduced in 1972, after the death of Master Irineu, is composed of blue trousers, a white buttoned-up shirt and a blue tie for men, and by a blue pleated skirt and a white blouse for women. The white uniform was introduced by Master Irineu in 1936 (though it has gone through changes over the years) and bears significant similarities to the uniform used in other Brazilian traditions such as the festival of St. Gonçalo which was prevalent in Irineu’s homeland of Maranhão (Labate and Pacheco, 2004; Dawson, 2013, 67). The male version of the white farda is composed of a white suit, with green stripes and a tie. The female white uniform is considerably more elaborate and includes a white pleated skirt or dress overlaid with a shorter green skirt signifying the forest and a long-sleeved blouse. Two green ribbons criss-cross the front of the white blouse, and a tiara or crown is worn on the head in allusion to the Queen of the Forest. Finally, an assortment of colorful ribbons (*alegrias*) is hung from the shoulder (Dawson, 2013, 67).

With its buttoned-up shirts, dark blue ties, glittering tiaras, and colorful ribbons the SD ritual uniform lends a reassuring air of respectability, order, symmetry and cleanliness to the ritual. Tramaachi notes the prevalence of practices of ‘beautification’ in entheogenic ceremonies exerting strong influence on set (Tramacchi, 2004, 133). Similarly, in the SD, the clean, respectable, uniformed attire acts to reassure and inspire trust in oneself and others. A reassuring sense of order is more easily inspired by members wearing a respectable attire than it would have been by members wearing rags. The bright, colorful glittering elements of the white uniform also serve to support the production of elating, transcendent experiences.

Both types of uniform conform with a broader prescription of ritual colors which might be termed the daimista palette. An interesting feature of SD space, namely, is the sanctification of specific colors (green, blue and white–representing the earth, sky and astral realm) and the prohibition of other colors (red and black). This choice of colors is evident in the daimista uniforms and in the SD flag (utilizing the same colors, as specified in a popular hymn by Padrinho Alfredo (Melo Sebastião, 7)). Objects entering ritual space should ideally conform with this palette preferencing specific colors and proscribing others.

The universal nature of color preferences has been amply debated by scholars (Park and Guerin, 2002; Taylor et al., 2013). Some observers have argued that certain colors, evolutionarily associated with life-supporting natural phenomena (e.g. vegetative green, blue associated with sky and water), have universally soothing effects (Wexner, 1954; Mahnke, 1996). Others argue that color preference is culturally constructed (Taylor et al., 2013). Whatever position one takes on this point, all sides agree that cultural color preferences exist, whether based in nature or in cultural symbolical matrices. The cool, nature-related colors of the SD palette, symbolically associated with the earth, sky and the astral, therefore become suffused with inalienable meaning. The use of these colors in SD uniform and ritual space can be construed as another way in which visual cues are employed to produce specific effects.

### Musical Components

Same as visual acuity, musical acuity and auditory appreciation too are enhanced under the effects of psychedelics (Hoffer, 1965; Weber, 1967; Kaelen et al., 2015; Barrett et al., 2018). The central role of music in guiding experiences with psychedelics has been noted and explored by numerous investigators (Bastos, 2007; Barbec de Mori, 2015; Labate et al., 2017; Kaelen et al., 2018).

Music takes a central role in psychedelic therapy. Kaelen et al. find a correlation between patients’ openness to the music and the occurrence of peak experience as well autobiographical insight. They conclude that the therapeutic outcomes of psilocybin treatment are in fact a product of the combined effects of drug and music on subjective experience (Kaelen et al., 2018). Music also assumes a central role in numerous types of entheogenic ritual settings (Tramacchi, 2004; Barbec de Mori, 2015). Bastos, describing the role of music in the indigenous societies of Iowland, South America, describes music as a “centripetal force enabling the convergence of the visual, olfactory and other kinds of discourse composing the rites” (Bastos, 2007, 5).

SD is often described as a musical doctrine and the central role of music in the SD has been explored by various authors (Rehen, 2007; Labate and Pacheco, 2010; Labate et al., 2017). As Labate et al. put it:

In Santo Daime, the ‘hymns’ are the main conductors of religious ritual. Memory, emotion, language, and cognition are all mobilized in the sonic experience of ayahuasca. Without music, there is no ritual, and no guided experience of ecstasy. In sum, music is a technology that permits intersubjective communication between persons, species, and peoples. (Labate et al., 2017, 103–104).

Elsewhere they add:

Music provides structure to rituals, creates narrative, activates deep emotions, produces religious ecstasy, and permits spiritual transcendence; it invokes collective memory and tears down and rebuilds notions of time and space, creating the experience of a self-evident, intangible truth. In other words, music itself creates the religious universe. (Labate et al., 2017, 102–103).

The musical roots of SD were explored by Labate and Pacheco, who find relationships between the hymns and calls (chamadas) of SD and blessings (benditos) performed at catholic ceremonies in Brazil, and with afro-brazilian traditions from the state of Maranhão, (Labate and Pacheco, 2004, 2010) though Dawson regards this evidential chain as speculative. (Dawson, 2013, 12).

Principally SD music is rather generic in nature. It is sung in unison and led by three types of basic rhythms (accompanied by respective dances): the march, the waltz and the mazurka, two of which have their origins in popular Catholic festivals (Dawson, 2013, 60). The principle musical instruments are the guitar, and the maraca, with the occasional addition of other instruments. The singing of hymns is performed by all ritual participants, though often led by a female singer (*puxadora*) proficient in the minute nuances of correct performance.

Despite its plain nature, SD music has often been described as particularly beautiful and enchanting (Shanon, 2002). Its attractiveness no doubt benefits from the effects of daime. The unison character of the music contributes to a feeling closeness among the participants, united in their efforts to perform harmoniously (Tramacchi, 2004). The percussive sounds of the maraca, additionally, have been associated with the induction of synesthesia and other mind-altering phenomena (Johnston, 1977).

As the ritual progresses one readily observes how music guides ceremonial climaxes and nadirs. Some particularly enthralling hymns may swiftly raise sound and energy levels leading the work to new heights, while other slower parts (e.g. waltzes) can sometimes drag and bring about a certain heaviness.

In a sense, one could view this arrangement of ritual music as a programming for individual and collective visions. Dobkin de Rios and Katz describe the role of music in drug experiences as ‘Jungle Gym’ for consciousness. They argue that music “provides a series of pathways and banisters through which the drug user negotiates his way” and that the “companionship of music to the hallucinogenic drug experience functions almost like a computer’s magnetic tape. It can instruct the calculating machine in a particular course to follow” (Dobkin de Rios and Katz, 1975, 208–209).

This description of the role of music in hallucinogenic experiences as similar to a computer program instructing the processor to perform specific activities and enter specific states is reminiscent of Metzner and Leary’s assertion that through set and setting, the psychedelic experience could be programmed (Metzner and Leary, 1967). Since, unlike many ayahuasca rituals, the order of hymns in SD rituals is predetermined, this analysis invites an interpretation which views the different works in the SD repertoire as types of computer programs similar to those described by Dobkin de Rios and Katz, leading the listeners through diverse internal landscapes and mindstates. Concentration works, healing works, and mass can therefore be metaphorically viewed as calculated programs for the induction of specific effects in ritual visitors.

### Olfactory Dimensions

The crafted use of perfumes and odors for directing experiences with psychedelics has been documented by anthropologists (Luna, 1986; Beyer, 2010). The effects of such interventions seem to be primarily context dependent. Much like the research on color perception mentioned above, research on the effects of odors and scents on human cognition, emotions and mood has led to rich but inconclusive findings. Here too, a review of the literature finds that contrary to popular claims of aromatherapy, scent preferences and effects are not universal. Rather, they are mediated by the cultural significance awarded to different odors and by personal preference (Herz, 2002, 2009). Research has found that the mood effects of odors dependend first and foremost on the use of suggestion. Thus, the suggestion that an ambient odor is relaxing produces relaxing effects, while the suggestion that a certain ambient odor is stimulating produces stimulating effects (Campenni et al., 2004).

These findings are relevant to thinking about the role of scent in SD rituals, which, according to Labate and Pacheco, might have roots in the traditions of Irineu’s homeland Maranhão (Labate and Pacheco, 2004). At specific points during the ritual, the work is halted, and guardians carrying censers or sticks of incense enter the floor, blowing fragrant smells into ritual space to the accompaniment of music. Some participants stand up with their arms stretched beside their body, signaling the guardians to approach them and carefully blow incense smoke over their bodies to purify of evil influences.

Importantly, the use of incense in SD also borrows from Brazilian Umbanda religion, which makes elaborate use of diverse types of incense mentioned in the popular Umbanda hymn (Umbanda Defuma com as ervas da Jurema) incorporated into the SD repertoire and performed alongside the blowing of incense smoke in the salon (Umbanda Defuma com as ervas da Jurema). The hymn makes reference to a variety of plants including the mimosa plant (*jurema*)*,* rue (*arruda*)*,* guinea (*guiné*)*,* benjoim (*benzoin*)*,* alercrim (*rosemary*) and alfazema (lavender). While the particular types of incense used may differ across churches, the culturally significant associations of these scents within the SD context charges them with a unified, coherent significance of cleansing and purification that synergizes with other ritual elements to enhance ritual efficacy.

### Kinetic and Proprioceptive Experience

A few short words on the topic of bodily movement and posture in SD rituals. As noted above, SD rituals are divided into two types: dancing works and seated works.

**Dancing.** SD rituals include three main types of ritual dances corresponding to the three musical rhythms of SD (march, waltz, mazurka) and consisting of repeated balanced movements from left to right. Dancing works typically consist of between eight to twelve hours of continued, festive, synchronized dance. The use of collective dance rituals for the induction of transformative experiences can be found across numerous cultures and shamanic traditions utilizing entheogens (Tramacchi, 2004). Tramacchi notes that “prolonged, vigorous dancing can be sufficient in itself to induce mood-altering biochemical changes and altered states of consciousness” (Tramacchi, 2004, 135). This is certainly true in the case of SD works, where dancing rituals continue relentlessly for entire nights and where participants reach states of extreme physical exhaustion. During a dancing work, participants might reach points of extreme fatigue where the idea of continuing for many more hours seems almost inconceivable, only to later become reinvigorated (by music, an additional cup of daime, or the general ebb and flow of ritual) and spend several more hours energetically dancing. Confronting one’s physical limits and overcoming them during an extended night-long work is often a key factor shaping daimista experiences.

**Seated.** Other non-dance works such as the concentration work, healing works, mass works etc., are conducted seated on a chair. Participants are instructed to sit up straight with their heads high and not cross their arms or legs (this is considered to close the body and block the flow of ‘energy’). Previous research found that ayahuasca promotes introspective reflection (Riba et al., 2001). Such psychopharmacological capacities are potentiated in the context of seated works. In view of research that demonstrates that body postures are associated with states of mind (Briñol et al., 2009; Constantineau and McLuhan, 2010; Veenstra et al., 2017), this respectful, upright body posture arguably fosters an active, composed, and reassured state of mind, which might be contrasted with, for instance, the more relaxed lying down postures common in many shamanic-type ayahuasca ceremonies, though it might, by contrast, also lead to feelings of constriction and discomfort (Importantly, SD rituals also include 'cure rooms' where one can lie down and rest).

### Skillset

“In order to learn/in this school of the lord/it’s necessary to have love/and pay attention/To what the teacher teaches in class/and what he writes on the board/and the homework to take home/Every student/knows it is an obligation/to visit school/in order to learn the lessons (…) For in this school/the teaching is spiritual/let us pay attention/so that we get our degree” (Melo Valdete, 16).

The notion of skillset is a relatively novel addition to the scheme mapping the extra-pharmacological factors shaping drug effects. It is discussed by Godasi who defines skillset as a set of cultivable techniques, strategies, and approaches for navigating experiences with psychedelics (Godasi, 2019).

The notion of skillset is notably implicit in the worldview of shamanism, where a long and arduous route of initiation is thought to awaken and develop certain qualities in the initiate (Macrae, 1992; Beyer, 2010). A similar process is arguably present in SD religion, and some scholars have indeed compared SD practices to a form of communal or collective shamanism where participants (collectively singing and dancing) all take part in shamanic flying and undergo shamanic initiation (Couto, 2004).

Remarkably, SD makes ample reference to its rituals as pedagogic activities that assist practitioners in cultivating traits that are valuable for navigating ritual space and can thus be construed as part of a skillset. SD hymns make repeated reference to the need to cultivate mental qualities of strength, patience, love, and joy. Most prominently SD hymns stress the need to stay firm and hold one’s place even under challenging circumstances, when one is overwhelmed by physical and mental challenges brought about by the consumption of daime.

Physically, firmness is needed to withstand common experiences of nausea, weakness and fatigue. Mentally, staying firm means not letting one’s mind get caught up in negative thought patterns such as shame, remorse, anger or fear but rather orient oneself toward compassion and love.

More broadly still, the concept of *firmeza* (firmness) refers to the ability to gracefully withstand suffering, pain, frustrations and humiliations, while remaining focused on beauty and love (Melo Sebastião, 3). It is a spiritual ideal reminiscent of the ideals of stoicism or the Buddhist ideal of equanimity (Gethin, 1998). SD rituals involve frequent sensations of physical discomfort which, combined with the demands of prolonged and collectively coordinated ritual, challenge the daimista. Since these challenges of ritual are considered inseparable from its cleansing, elevating qualities, the emergent daimista ideal is that of a balance: gracefully receiving the often-fierce presence of divine light while staying firm and not running away from the pain. This is described as “remaining composed within the light” (Melo Alfredo, 159).

Significantly, as noted by Dawson, the frequent hymnal admonitions to ‘stay firm,’ ‘remain in your place,’ ‘pay attention,’ and 'firm your thoughts" are conducive not only to the development of participants’ personal skillset and merit, but also to the successful performance of the collective ritual (Dawson, 2013, 65). Equally important is that skills cultivated and honed in ritual space carry a significance that extends beyond the ritual and into everyday life. By keeping their wits in the testing moments of entheogenic spiritual battle, participants are assumed to cultivate more general qualities that help them stay firm facing the challenges of everyday existence.

This notion of SD doctrine as a pedagogical enterprise is present in the characterization of SD as a school, and its members as pupils, visiting lessons and acquiring skills and degrees (Cemin, 2004; Albuquerque, 2012). SD is here understood as a type of entheogenic *dojo,* a spiritual school (*escola espiritual*) where *daimista* pupils/soldiers acquire the rudiments of spiritual education/warfare. This idea recurs across many of the hymnaries. Several hymns refer to the ABC, describing members as learning the ABC of spiritual education (Melo Rita, 25; Melo Sebastião, 28; Melo Valdete, 16; Silva O, 17). The repeated reference to learning the ABC delivers a point about the incipient nature of human attempts to approach spiritual perfection, but also lays out a path of continuing, progressing curriculum, thereby setting it apart from haphazard uses of psychedelics, and aligning it with traditions where hallucinogenic use is considered an evolving practice and expertise.

Importantly, SD offers its members diverse types of sites and skills in which to specialize and grow. Daimistas may choose to assume varied types of roles associated with varied strengths and qualities. These include the role of a musician, a singer, a church leader, a daime server, a decorator of ritual space, or a guardian to give a few prominent examples. Each role offers different types of challenges, curricula, and skills to be perfected. Each role also associates with different types of set and setting and profoundly different types of experiences. By performing a role, a daimista may connect to a broader archetype or mission associated with that role and embody its characteristic features and qualities, so that a guardian in the salon becomes truly a guardian of the divine doctrine. “Ritual performances have this function” writes Leach “they momentarily make explicit what is otherwise a fiction.” (Leach, 1964, 16).

Importantly, different churches offer different routes of education and development for daimistas. For instance, small, non-official SD congregations (*puntos*), where each member may be cardinal for the successful execution of ritual, allow greater opportunity (or demand) for members to take on responsibilities and acquire first-hand knowledge of the fundamental machinations of ritual. In larger church, by contrast, key positions are often filled up, and members may spend years assuming more of a backseat stance to ritual.

Simultaneously, membership in a larger daimista community confers other educational opportunities: encountering SD dignitaries traveling between major churches, learning the finely nuanced art of SD ritual from well-seasoned daimistas, and being able to attend a larger number of works and to sing and become acquainted with a broader selection of hymn books–opportunities typically lacking in smaller communities.

### Social Setting - Community

Social environment plays a large role in shaping experiences with psychedelics(Hartogsohn, 2017). One study by Hyde found that changes in the attitude of medical staff towards a study’s participants greatly impacted experimental results, exacerbating difficult experiences when staff was unfriendly, and mitigating them when staff was friendly (Hyde, 1960). A familiar, friendly environment produced resulted in pleasant effects, while an unfamiliar, unfriendly environment exacerbated netgative effects–an observation that stands to reason, and is further corroborated by a comparative examination of the trajectories of hallucinogenic research (Hartogsohn, 2020).

The central role of social environment is also discussed by anthropologists who regularly invoke Turner’s concept of *communitas* to describe the manner in which socially sanctioned use of hallucinogens leads to greater social cohesion and solidarity (Slotkin, 1956; Myerhoff, 1975; Tramacchi, 2004). Applying this idea to the case of SD, MacRae argues that SD rituals “move social life and, consequently, society, towards order and structure” (Macrae, 1992, 4).

SD rituals are indeed a strikingly social enterprise whose collective nature is thought to benefit all involved. As Dawson notes “collective ceremonial practice … furnishes a return on subjective cultic action far greater than that ordinarily available to an individual working in ritual isolation” (Dawson, 2013, 75). Collective singing, and the psychotropic effects of daime both act as powerful stimulants for interpersonal ties and community building (Cemin, 2010). A communitarian aspect figures prominently in the stories of Master Irineu’s Alto Santo community, and Padrinho Sebastião’s communitarian community. The *Decree of Master Irineu*, a foundational text of daimista faith, acknowledges the communitarian nature of daimista congregations and specifies the responsibility of the community to care for its sick members (Serra, 1970). Importantly, daimista language employs what MacRae terms “familial ideology,” (Macrae, 1992, 3). which regularly describes the members of SD as a brotherhood (*irmandade*) or family (*familia*)*,* further enhancing the idea of close relationships between ritual participants (See for instance Maria, 22).

While the newer urban-professional constituency of SD described by Dawson doesn’t follow this communitarian ideal in full (Dawson, 2013), SD churches nevertheless often retain strong communal aspects. Communal ties are supported by the oft-observed ability of classic psychedelics to support social connection (Gearin, 2016; Forstmann et al., 2020) as well as by contextual factors: a shared interest in SD and by the prolonged association and cooperation required of members to sustain church activities. SD churches are often active for years and decades, and unlike some 'freestyle' type ayahuasca groups, where the identity of ceremony participants varies from one occasion to the next, SD church members usually frequent the same church for extended periods–sometimes throughout entire lives. This ongoing commitment to a community allows a solid, long-term basis for friendships to cohere.

The tight-knit character of SD communities nevertheless also presents distinct quandaries. The challenges and disappointments of community life are mentioned in many memorable hymns from SD’s foundational figures, who proclaim their disappointment with their flock and admonish their communities to bicker less and respect their brethren (See for instance Maria; Melo Sebastião; Serra). When communal relations become defunct the long-term nature of SD churches can lead to communal conflicts and rivalries that linger for years without reaching resolution, which, in some cases may lead to detrimental effects for set and setting, or even to the breakup of communities.

The intense, viscerally challenging nature of SD ritual acts as an additional catalyst for communal intimacy. SD relationships are welded and tested in extreme battle conditions, where all are called to close the lines. In a sense they resemble the type of war-camaraderie that is formed between soldiers who have gone to battle together. Such comparisons might first seem indulgent, however the daimista battlefield worldview might indeed prove useful to understanding the intensely binding nature of altered-states battle in the salon, where members enter into liminal states and work together as a battalion to complete a multiple-hour march that lasts throughout the night, and in which members witness each other in extreme (and extremely intimate) states of nausea, purging, helplessness and desperation but also joy, ecstasy and bravery. After battling together for entire nights to achieve victory, members often feel an intensified sense of camaraderie and intimacy that accords with previous research demonstrating that extreme rituals promote prosociality (Konvalinka et al., 2011; Xygalatas et al., 2013).

Finally, strong interpersonal links are also mandated, at least for the core members, by the organizational requirements of SD church activities. A lot needs to happen for a salon to run smoothly through long arduous works, and for a church to keep going for years. Strong interpersonal foundations are often required to keep a church going through the oft-herculean challenges of organizing and orchestrating long and frequent hymnary works, or entertaining visiting entourages consisting of multiple musicians and dignitaries who need to be boarded, fed and shown around.

The long-term nature of relationships within SD communities supports the creation of long-term social setting, built gradually over years of joint ritual work. This long-term social setting tends to be dynamic, changing and evolving across the years. Importantly it gives rise to intriguing implications regarding the mind-altering properties of social relationships themselves.

To understand this phenomenon, one might invoke the concept of contact high, widely known among users of hallucinogens (Tart, 1971; Olson et al., 2020). The concept generally refers to the phenomena in which individuals who have not taken a psychoactive report psychoactive effects supposedly induced by social contact with another individual that *has* taken a psychoactive. Recently, this phenomenon was the subject of a paper which explains it by referring to mechanisms of classical conditioning, positive expectations, emotional contagion, and social modelling (Olson et al., 2020).

Contact high can then be thought of as a kind of resonance or mirroring that allows for the transmission of at least some aspects of altered states to certain proximate others. What makes the case of long-stand entheogenic communities, such as SD communities, particularly interesting is the continuous nature of involvement these groups have with altered states of consciousness. In such long-term tight knit communities, the experience of contact high can be induced before the first cup of daime has even been served. The encounter with a group of individuals strongly associated with mind-altered states within a familiar ritual space where one has repeatedly and communally entered such states are often enough to conditionally reproduce these past states of mind associated with the same context, a phenomena readily predicted and explained by the conditional nature of placebo response (Stewart-Williams, 2004).

In some cases, the attractive force of the social can become greater than that of daime itself. Some daimistas might continue visiting works even when their faith or desire to drink daime are attenuated, solely in order to reconvene with dear friends. This has the potential of profoundly complicating the relationship between ritual, social contacts, and psychoactive sacrament. Sometimes a person might wish to desist from drinking daime, but still wish to associate with the social matrix. This markedly communitarian aspect of the SD set and setting renders the SD experience exceedingly relational and distinguishes it from individualistic types of set and setting, such as those of psychedelic therapy.

### Integration

A final aspect of the extra-pharmacological set and setting which needs to be addressed is the subject of post-session integration. The subject of integration has lately received growing attention in scholarly and popular discourse on psychedelics (Westrum and Dufrechou, 2019; Watts and Luoma, 2020; Godasi, 2019). While scholarly work about integration is still scarce, the concept has been around for some time, under the banner of support (Fadiman, 2011), or Matrix, which Eisner defines as the environment to which a person returns after a psychedelic experience, and the degree to which that environment allows them to integrate and make use of any insights they may have gleaned (Eisner, 1997).

In terms of the living conditions to which daimistas return after rituals, the integrative framework SD communities offer their members is quite varied. Early SD churches often fulfilled ideals of closeness and proximity, allowing ample space for integration. Such arrangements have long since ceased to be the norm, and the types of living situations to which daimistas return after participation in a SD work are as varied as the many communities and the different circumstances of their members.

Nevertheless, SD religion does offer certain fundamentals for the processing and integration of the entheogenic experiences. After concluding the work, daimistas often (depending on church and occasion) stay around ritual space for long hours, excitedly discussing the nights events. This allows ample opportunity for discussion, examination and reprocessing of the events of the work. Meanwhile, SD also presents an interpretative framework used to make sense of members experiences by the employment of concepts such as *miracão* (vision) or *atuacão* (uncontrolled incorporation of a spirit) that are used to make sense of transpired events. The divine status accorded to SD ritual leads to favorable interpretations of the experiences within the ritual, reinterpreting even difficult events as potentially ‘healing,’ ‘purging,’ or otherwise beneficial. Thus, daimistas are quite proficient in reinterpreting entheogenic experiences so that difficult, excruciating experiences are reframed as healing, revealing and ultimately positive in the grander scheme of things.

### When Set and Setting Goes Wrong

SD ritual presents us with an elaborate example of the organization of contextual elements in conjunction with a psychedelic for the induction of positive experiences of healing, ecstasy, and transformation. Nevertheless, it also presents a set of typical complications.^13^

No arrangement of set and setting is perfect for all or under all circumstances. An environment that is illuminating and inspiring for one can prove exasperating and frustrating for another, depending on the individual and their circumstances. For instance, issues of identity might intervene to make the SD set and setting less than perfect, including, as discussed above, for LGBTQ individuals, or for non-Christians who might be upset by frequent references to Christian figures.

In this section, based on personal observations and ongoing informal conversations with daimistas, I wish to address some potential trappings of the set and setting of SD–the ways in which SD setting can go wrong. Often these trappings mirror the very strengths of the SD setting, demonstrating how these strengths can turn into weaknesses once taken to the extreme.

**Over-exertion and Spiritual Injury.** The ability of daimistas to devote themselves fully and unconditionally gives the SD work much of its unique power, but it can also have its dark side.

As mentioned, daimistas can be incredibly devoted to serving their congregation in the spiritual battlefield of ritual. SD hymns urge participants to “show their value” (*mostrar o seu valor*) (through their performance in the ritual, among other things).

Unfortunately, sometimes SD soldiers can become too zealous–committed to the point of injury: for example by continuing to dance despite an aching knee, or continuing their guitar playing despite shoulder pain–because this is what ritual calls for. If you’re a strongly motivated soldier in the army of Juramidam and you don’t watch it, you might get injured in battle.

This injury might occur at the physical level, but it can also occur on a mental level. When daimistas push themselves too hard, obliterating their needs and over-exerting themselves, they can incur what we might call a spiritual injury, leading to exhaustion, a mental ‘overdose,’ and the need to distance oneself from rituals. Being too keen a warrior can sometimes end up sabotaging one’s path. This is one danger daimistas need to be conscious of.

**Shame, guilt and rebellion.** The central role of order in SD ritual implies the possibility of unease for those who feel constrained by order (Couto, 2004, 397–398). Moreover, while the efficacy of SD ritual derives from its highly structured nature, the structured character of SD ritual can become counterproductive when it is followed too closely and rigidly.

SD works are ideally orderly and well-held. The sense of carefully tuned order and harmony created by the music, aesthetics and structured nature of the SD ritual breeds a feeling of security and allows participants to approach transcendence. However, a strict adherence to ritual protocol can feel constricting to some (Macrae, 1992, 4). Over-adherence to order might lead to ossification and act as a destructive force for the current of the work.

Allowing elements of disorder to express themselves can thus prove vital. Labate et al. note the ‘hyper-realism’ (or hyper-orthodoxy) of European churches whose ceremonies seem “more orthodox than in the native Brazilian ceremonies” (Labate et al., 2017, 107). Western daimistas visiting Brazil for the first time are often surprised to find that Brazilian SD churches are not quite as strict about fulfilling ritual guidelines as those in the west. The more flexible, less orthodox, nature of these churches can lead to a superior spiritual current (alternatively, it might stymy the power of ritual). Excessive order in the salon can be as counterproductive as disorder.

Another limitation of excessive ordering is that it can easily lead to feelings of guilt, inadequacy and shame in those individuals who struggle to conform with expected levels of order and discipline (Weinhold, 2007). Psychedelics are able to evoke challenging high-anxiety experiences (Carbonaro et al., 2016). An overly regulated setting can often create feelings of constriction and anxiety about ritual excesses and inaccuracies such as singing out of tune or too loudly, moving too noisily or dancing too fervently. Alternatively, in some individuals, it might lead to feelings of resistance toward doctrinal rules, referred to as *rebeldia* (rebellion) in the hymns. Such challenges are less likely to appear for instance in freestyle ayahuasca ceremonies where ordinances are less strict. SD churches therefore need to strike a balance between structure and order to keep the *current* flowing smoothly and harmoniously.

**Encountering the Ego.** Psychedelics are often described as ego dissolving, ego-eradicating pharmacological tools (See for instance Lebedev et al., 2015; Nour et al., 2016). The importance of diminishing the ego is also mentioned by several prominent SD hymns. A hymn by Padrinho Valdete states “I don’t drink daime/to aggrandize myself” (Melo Valdete, 14). Another Hymn by Alex Polari speaks of “breaking the ego” (Alverga).

Nevertheless, things are not quite as simple, and psychedelics also been used by narcissistic personalities for ego-amplifying purposes.^14^ Psychedelics are often described as amplifying mental phenomena (Hartogsohn, 2018a), and may under some circumstances also intensify and augment egoic structures.

While SD ritual aims to diminish the ego, the architecture of SD ritual space, where participants spend hours facing each other may also amplify the perception of ego. And while participants are encouraged to gaze inwards and not stare, SD is a remarkably social enterprise. During moments of fervor and ecstasy in the salon, eyes are often wide-open, and gazes tend to meet. Since the feeling of being observed easily leads to greater self-consciousness (See for instance Harris, 2014, 110–115), the juxtaposition of the performative aspects of SD, the architectural arrangement of space, and the potentially ego-enhancing effects of psychedelics, may end up not obliterating but augmenting an ego preoccupied with questions such as how one is heard, viewed, thought of etc. ^15^

The doctrinal advice in such cases would be to let go of such thoughts and concentrate on the hymns and their messages. A daimista’s ability of following this advice depends on their skillset.

As I have attempted to show, SD ritual prescriptions do not by themselves guarantee a positive outcome. A certain balance needs to be met between fluidity and rigidity, between wanting to be a good soldier and the need to care for oneself.

These challenges of set and setting are typical to SD’s ritual structure, yet they are not unavoidable. Though SD ritual setting can go wrong in several ways, most daimistas would argue that the proper way of performing the work should always be in line with the cultivation of the daimista’s physical, mental and spiritual health, so that, in principle at least, informed and conscientious members should avoid the traps of set and setting. In practice, finding the golden path can be tricky, so that navigating between the *Scylla* and Charybdis of freedom and rigidity, dedication and self-care can be a lifelong endeavor.

## Conclusion

As I hope this paper has clearly shown, drinking daime is much more than just drinking ayahuasca. The effects of ayahusca are profoundly mediated through complex layers of intentions, expectations, visual, auditory, and symbolic environments, social and cultural systems, etc. These various contextual factors comprise a rich cultural apparatus that serves to mitigate harms and facilitate social and personal benefits (MacRae, 2009; Blainey, 2015). Such contextual factors produce a distinct form of experience, that is often markedly different from the experiences produced by other contextual environments, e.g., those of neo-shamanic ayahuasca rituals, or other ayahuasca religions. While a full exploration of such variations is beyond the scope of this paper, future research might produce comparative works examining the modulation of ayahuasca effects under different contextual environments.

While previous work has amply described the SD ritual, this paper contributes by systematically relating diverse aspects of SD ritual with their characteristic effects on participants in SD rituals. It offers a first of its kind analysis which contextualizes SD ritual using insights from the literature on set and setting and the social and cultural history of SD. Furthermore, it explores the crucial role of elements that have so far been overlooked such as the role of skillset, integration, identity, the sacrosanct status of ritual and daime, religious calendar, olfactory and synesthetic elements, among other things. This examination could unquestionably be further extended to include additional details and dimensions of SD ritual and culture, yet my hope is that it nevertheless provides the reader with a useful, succinct yet rich example of the way a ritual environment can be analyzed to uncover its characteristic biases and modes of influence. While the formalized, generalizable structure of SD rituals facilitated the performance of this analysis, its contents reveal the many intricate ways in which experiences with psychedelics are intimately informed by context.

## Data Availability

The original contributions presented in the study are included in the article/Supplementary Material, further inquiries can be directed to the corresponding author.

## Footnotes

1 The number of SD practitioners globally is assumed to be in the tens of thousands, and SD churches are spread throughout the globe (Labate et al., 2017).

2 For an extended description of these developments see Hartogsohn, 2017.

3
Dawson, 2013, 35. The similarities and crucial differences between these lines are discussed extensively by Orgad (Orgad, 2012).

4 Primarily concentration, healing works, mass, and hymnary works, excluding special events such as birthday works, marriages, funerals, and feitio works.

5 For a discussion of skillset see Godasi, 2019. Importantly these diverse elements often intertwine so that a thoroughly hermetic distinction between them is often impossible. This, however, matters little for the purposes of this discussion.

6 (Moreira and MacRae, 2011, 142–174, 305–333). Remarkably, Irineu led several types of works that are not in common use in SD today such as table works and chamados.

7 Some of the main points of contention regard the ritual use of the cannabis plant and the practice of spirit mediumship within the ICEFLU line. See Orgad, 2012.

8 (Melo Alfredo, 18).

9 Dawson provides an extensive list of the rapidly growing network of popular influences on daimista faith. See Dawson, 2013, 37–38. Elaborate analyses of the symbolical meaning of daimista concepts can be found in Orgad, 2012 and Groisman, 1991.

10
Labate et al., 2017. This is particularly true in the case of dancing works, and somewhat different in sitting works, which include stronger elements of surrender and contemplation.

11 These zones are further divided into three sections according to age.

12 Though height, age and marital status are also important in this regard.

13 I define complications here as the induction of states of mind that contradict the professed values of the doctrine.

14 One famous example is the use of LSD by American criminal and cult leader Charles Manson.

15 The question of subjective preoccupation with the self is discussed by Dawson, 2013, Ch. 3.
